# Exploratory cluster analysis of self-reported adverse events associated with antidepressants using Gower distance and partitioning around medoids: a single-center cross-sectional study

**DOI:** 10.3389/fpsyt.2026.1916987

**Published:** 2026-09-17

**Authors:** Qian Zhai, Fang Yan, Han Qi, Ling Zhang, Gang Wang, Lei Feng

**Affiliations:** Beijing Anding Hospital, Capital Medical University, Beijing, China

**Keywords:** adverse events, antidepressants, cluster analysis, Gower distance, partitioning around medoids

## Abstract

**Background​:**

Antidepressants are first-line pharmacological interventions for depressive disorders, with globally increasing prescription volumes. Associated adverse events (AEs) represent the primary reason for treatment discontinuation. Existing studies mostly limit analysis to simple stratification by organ system or severity, while exploratory subtyping grounded in multidimensional clinical features of AEs remains scarce.

**Objective:**

This study aims to overcome the limitations of traditional single-dimensional analyses by integrating multidimensional data—including demographic and sociological​ characteristics, clinical disease severity, and AE-related information—to conduct exploratory cluster subtyping at the patient level.

**Methods:**

This study is an exploratory secondary analysis of a single-center, retrospective, recall-based cross-sectional survey on antidepressant adverse events registered with the Chinese Clinical Trial Registry (ChiCTR2500111836). It included 500 patients (905 AE episodes) self-reporting AEs within the past year at Beijing Anding Hospital (April 2025–January 2026). Unlike the original survey’s descriptive aim, this analysis identified heterogeneous patient subtypes: one AE per patient ID ensured independence; the Gower-PAM algorithm clustered 23 variables; and bootstrap-derived Adjusted Rand Index (ARI) values plus an all-event sensitivity analysis confirmed robust subtyping stability.

**Results:**

K = 2 was the optimal clustering solution (silhouette coefficient = 0.219; mean ARI = 0.916). Cluster 1 (42.2%) was characterized by early onset, high burden, and discontinuation-prone​ features, with moderate-to-severe AEs in 65.9% and a discontinuation rate of 75.8%. Cluster 2 (57.8%) was late-onset, low-burden, and high-tolerance, with mild AEs in 75.4% and a discontinuation rate of 16.6%. Significant differences were observed between groups in social support, work stress, and improvement of depressive symptoms.

**Conclusions​:**

This study identified two AE subtypes, revealing a concomitant pattern of “disease burden–psychosocial resources–AE tolerance,” providing exploratory evidence for risk stratification. Limited by the single-center retrospective design and self-report bias, validation in future prospective multicenter studies is needed.

**Clinical trial registration:**

https://www.chictr.org.cn/bin/home, identifier ChiCTR2500111836.

## 1 Research background

Antidepressants are a first-line intervention for depressive disorders, and global prescription volumes continue to rise (1, 2). Recent real-world prescription studies have demonstrated a marked increasing trend in antidepressant prescribing, with notable subgroup differences across age, sex, and drug classes—female patients accounted for up to 66.11%, prescription rates increased with age, and significant differences were also observed in the distribution of drug classes [including tricyclic antidepressants, selective serotonin reuptake inhibitors (SSRIs), serotonin-norepinephrine reuptake inhibitors (SNRIs), and atypical antidepressants [the broad classification adopted in that study)] between sexes (3). Against this background, clinical monitoring of antidepressant-related adverse drug reactions (ADRs) is particularly important. ADRs are common in clinical practice, encompassing gastrointestinal discomfort, weight gain, sexual dysfunction, sedation, and insomnia, among others. These may reduce medication adherence and, in severe cases, lead to treatment discontinuation or switching (4, 5). Existing data indicate that antidepressant discontinuation rates due to ADRs vary substantially across different agents, reaching up to over 50% at the highest, and patients exhibit heterogeneity in adverse event profiles, severity, and clinical outcomes (5).

Pharmacoepidemiology and psychiatric research are progressively shifting from “single-disease description” to “patient heterogeneity analysis.” For instance, latent class analysis based on biomarkers has identified homogeneous subgroups with distinct metabolic health profiles among patients with depressive and anxiety disorders, supporting the rationale for data-driven patient subtyping (6). In the field of adverse event research, previous studies have employed cluster analysis of concurrently reported adverse events to identify natural groupings, thereby characterizing such heterogeneity (7); recent investigations have also focused on patient-reported antidepressant adverse events and their impact on daily functioning (4). Nevertheless, existing studies remain limited in integrating multidimensional clinical characteristics for patient subtyping: most analyses focus solely on individual events or are restricted to stratification by organ system or severity. There is a relative paucity of studies that systematically delineate subtypes by incorporating multisource information at the patient level, including demographic characteristics, clinical phenotypes, prior risk factors, and treatment discontinuation outcomes.

Compared with passive surveillance based on pharmacovigilance databases, patient self-reports more directly reflect medication experiences and yield granular data on the timing, severity, and management of adverse events (4). Simulation studies on clustering of mixed data types have provided methodological references for clinical data analysis; the Gower dissimilarity combined with the Partitioning Around Medoids (PAM) algorithm has been proposed and validated for clustering mixed-type data (8, 9). Against this background, this study is an exploratory secondary analysis of data from a single-center, retrospective, recall-based cross-sectional survey on antidepressant adverse events registered with the Chinese Clinical Trial Registry (ChiCTR2500111836). Using the Gower dissimilarity–based PAM algorithm, we performed patient-level cluster analysis integrating multidimensional clinical characteristics to explore potential classification patterns of self-reported antidepressant-related adverse events.

## 2 Methods

### 2.1 Study design

This study is an exploratory secondary analysis of a single-center cross-sectional survey dataset on antidepressant adverse events registered with the Chinese Clinical Trial Registry (ChiCTR2500111836).

Original study background:​ The original cross-sectional survey was independently designed and conducted by our team. Following ethics approval on March 28, 2025, the registration process was initiated. Due to a backlog at the registry center, final review and registration were completed on November 6, 2025, with no changes made to the original protocol or final analysis plan during this period. From April 7, 2025, to January 21, 2026, multidimensional data were collected face-to-face via structured questionnaires from 500 patients at Beijing Anding Hospital who self-reported antidepressant-related adverse events within the past year. The primary aim was to characterize discontinuation rates attributable to these adverse events, and detailed protocols are publicly available on the registry platform.

Positioning of this secondary analysis:​ Unlike the original study’s focus on discontinuation rates due to adverse events, this analysis centers on identifying heterogeneous patient subtypes. Using the Gower dissimilarity–based PAM algorithm, we integrated multidimensional data—including demographic and sociological characteristics, clinical disease severity, and adverse event–related information—to conduct exploratory cluster subtyping. The analysis plan was formulated after study registration to generate novel scientific hypotheses from the original survey data. Specifically, the clustering protocol was developed after primary data collection but was fully documented and finalized prior to executing any clustering algorithms.

Reporting guideline:​ This manuscript adheres strictly to the STROBE (Strengthening the Reporting of Observational Studies in Epidemiology) statement, specifically the STROBE-REC extension for observational studies using routinely collected data (**Supplementary Table 1**). The Methods section explicitly distinguishes between the original study design and the analytical strategy of this secondary analysis to ensure research transparency.

### 2.2 Participants

This study established stringent inclusion and exclusion criteria. Inclusion criteria: (1) aged ≥18 years; (2) receipt of antidepressant therapy at our hospital within the past year, including SSRIs, SNRIs, noradrenergic and specific serotonergic antidepressants (NaSSAs), serotonin antagonist and reuptake inhibitors (SARIs), melatonin receptor agonists, and aminoketone antidepressants; (3) self-perception of antidepressant-related adverse events; (4) literacy to independently complete self-report scales; and (5) voluntary provision of written informed consent. To avoid confounding, exclusion criteria comprised substance dependence, acute intoxication, intellectual disability, significant cognitive impairment, and severe physical illness.

A total of 556 candidates were screened. After excluding 18 refusals and 38 ineligible cases, 500 patients were enrolled, yielding an effective response rate of 89.93%. During the observation period, 905 adverse event occurrences were recorded. To prevent clustering bias from multiple event reports per patient and ensure sample independence, an event random sampling strategy was implemented: one event was randomly selected per patient ID, generating a clustering dataset of 500 independent samples. This approach retained patient-level information while circumventing pseudoreplication from repeated measurements, aligning with the independence assumption of the Gower dissimilarity-based PAM algorithm.

### 2.3 Research tools

This study employed a multidimensional assessment battery to systematically record participant demographic characteristics, clinical information, and self-reported antidepressant-related AEs. Data collection integrated standardized scales with a survey instrument developed for this study based on the existing literature.

#### 2.3.1 General demographic data collection form

Data were collected using a self-designed general information questionnaire, with items informed by the STROBE statement and the *Chinese Guidelines for the Prevention and Treatment of Depressive Disorders (2025 edition)* (10). Sociodemographic characteristics included sex, age, education level, marital status, monthly personal income, health insurance type, family support, work stress, and smoking and alcohol history. Family support was assessed via a single item based on the family well-being grading framework, categorized into four levels: no support, minimal support, moderate support, and complete support. This single-item measure demonstrated a test–retest reliability ICC of 0.76 in Chinese samples and showed significant positive correlations with total family functioning scores and subjective well-being scores (both *P* < 0.001) (11). Work stress was assessed using a single-item question from the Single-Item Stress Question (SISQ), validated by Arapovic-Johansson et al., adopting a 5-point scale (1 = none, 2 = low, 3 = moderate, 4 = high, 5 = very high) (12). Originally administered weekly via SMS to occupational populations, this item’s convergent validity has been verified against subscales of the Karasek Demand–Control–Support (JDCS) model and the Siegrist Effort–Reward Imbalance (ERI) model (13), with correlation coefficients ranging from −0.186 to 0.627; its test–retest reliability yielded weighted Kappa values of 0.804–0.868 (13). As ordinal single-item variables, Cronbach’s α was not calculated for these measures; their measurement properties were verified during a pretest. Clinical data included diagnosis, disease duration, comorbidities, and risk factors. Clinical diagnoses were independently assigned by two attending psychiatrists or above according to the Diagnostic and Statistical Manual of Mental Disorders, Fifth Edition (DSM-5) criteria (Kappa = 0.89) (14). The pretest showed a 100% item completion rate and a 94% item comprehension consistency rate.

#### 2.3.2 Adverse event reference table

For this study, we developed an *Adverse Event Reference Table* (**Supplementary Table 2**) as a terminological aid to standardize the naming of self-reported antidepressant-related adverse events (AEs). This table is not a psychometric scale and does not involve latent variable measurement; therefore, Cronbach’s α was not calculated. The reference table was adapted from the framework of the Patient-Rated Inventory of Side Effects (PRISE) (15), expanding its original 8-system classification to 12 organ systems based on the *Common Terminology Criteria for Adverse Events* (CTCAE v6.0) (16). The prototype of the PRISE was proposed by Rush and Asberg in 1999. Its formal multidimensional framework was systematically described by Wisniewski et al. (15) and was implemented across all visits in STAR*D* (17)*. Subsequent psychometric evaluations based on STAR*D samples reported that it comprises approximately 32 adverse event items, with an internal consistency Cronbach’s α of approximately 0.85. The Chinese version was culturally adapted through translation, expert review, and a pretest involving 20 patients. The terminology matching consistency rate was 92%, assessed during the translation–back-translation phase by a chief psychiatrist with >20 years of clinical experience. This assessment involved item-by-item comparisons among the original English version, the Chinese translated version, and the back-translated version, applying the criterion that clinical core meanings and severity grading descriptors must be free of ambiguity. The pretest with 20 patients further confirmed the absence of cultural or linguistic barriers to comprehension. Additionally, content validity evaluations by three chief psychiatrists (I-CVI > 0.78) supported the cross-cultural equivalence of the Chinese version.

#### 2.3.3 Antidepressant adverse event questionnaire

This questionnaire is a non-standardized instrument adapted from the framework of the Antidepressant Side-Effect Checklist (ASEC) developed by Uher et al. (18) and tailored to the characteristics of psychiatric clinical practice. It was used to structurally collect retrospectively self-reported information on antidepressant-related adverse events (**Supplementary Table 3**). As each item constitutes an independent module capturing heterogeneous characteristics—such as drug class, onset timing, and severity—rather than measuring a single latent variable, conventional psychometric analyses (e.g., Cronbach’s α) were not performed. Key item definitions: ① Symptom improvement was assessed with reference to the framework of the Clinical Global Impression–Improvement (CGI-I) scale (19). The original 7-point CGI-I rating was simplified to a 5-point self-report scale (marked improvement/moderate improvement/minimal improvement/no change/worse than before treatment), retaining the core logic of “improvement–no change–deterioration.” ② Adverse event severity was defined according to the Common Terminology Criteria for Adverse Events (CTCAE v6.0) (16), using a 4-level classification based on self-rated functional impairment: suspected/mild, mild (tolerable, no impairment), moderate (intolerable, impairs normal life/work/studies), and severe (extremely distressing, seriously compromises health and normal life/work/studies). The Chinese version was culturally adapted through translation, expert review, and a pretest involving 20 patients (terminology matching consistency rate: 95%). Content validity evaluation (I-CVI > 0.78) supported its cross-cultural equivalence.

### 2.4 Outcome measures

Primary outcome: Patient subtypes with antidepressant-related adverse events. Using multidimensional information—including demographic characteristics, disease severity, comorbidity burden, and adverse event profiles—unsupervised clustering algorithms were applied to partition the data and identify distinct patient subgroups. These subgroups demonstrated within-group homogeneity and between-group heterogeneity in medication tolerability, symptom severity, and psychosocial characteristics.

### 2.5 Statistical analysis

This study was a single-center, retrospectively recalled cross-sectional survey. All statistical analyses were performed using R (version 4.4.2). Statistical significance was set at a two-sided *P* < 0.05.

#### 2.5.1 Data preprocessing and variable definition

Initial data cleaning and variable preprocessing were performed on the raw dataset. For categorical variables, levels with frequencies <5 were collapsed into an “Other” category. To mitigate the influence of extreme values and skewed distributions on distance calculations, continuous and count clinical variables were uniformly rank-transformed prior to Gower dissimilarity computation. Descriptive statistics are presented on the original scale to facilitate clinical interpretation. A total of 23 variables were included in the clustering: 18 categorical variables (antidepressant class, organ system affected by adverse events, adverse event severity, degree of depressive symptom improvement, discontinuation status, sex, age, age at first onset, education level, housing type, family support, marital status, work stress, monthly personal income, health insurance type, alcohol use history, smoking history, family history of psychiatric disorders) and 5 continuous variables (number of prior risk factors, number of comorbid psychiatric disorders, number of episodes, number of suicidal behaviors post-diagnosis, number of physical illnesses). Details of variable preprocessing and Gower distance contributions are provided in **Supplementary Table 4**.

#### 2.5.2 Primary analysis: PAM clustering based on random sampling of events at the patient level

The primary analysis was performed at the patient level. Records were grouped by Patient_ID, and one event record was randomly selected per patient to construct an independent analysis subset of 500 cases. The Gower dissimilarity–based PAM algorithm was used to identify potential subtypes and generate patient-level subtyping results. The optimal number of clusters was determined by integrating the elbow plot and silhouette coefficient, with further validation using the Calinski-Harabasz (CH) and Davies-Bouldin (DB) indices. Clustering stability was evaluated via 1000 patient-level bootstrap resamples. After each resampling, PAM clustering was repeated; cluster labels were optimally aligned using the Hungarian algorithm, and the mean and standard deviation of the ARI were calculated as core stability metrics. Patient-level co-membership probabilities were also computed to provide a comprehensive assessment. Additionally, two-dimensional t-SNE was applied to visualize the high-dimensional Gower distance matrix.

#### 2.5.3 Sensitivity analysis: PAM clustering of all events

To verify the robustness of the sampling strategy, a sensitivity analysis was conducted without patient-level sampling. The Gower dissimilarity was computed directly on all 905 adverse event records (variables included event-specific characteristics and patient baseline characteristics replicated for each event), and PAM clustering was performed. Event-level cluster labels were then aggregated to the patient level by assigning the modal label per Patient_ID, yielding 500 patient-level labels.

#### 2.5.4 Methods for between-group comparisons

Continuous variables are presented as median (interquartile range) [M(Q_1_, Q_3_)] and were compared using the Kruskal–Wallis H test; categorical variables are presented as frequency (percentage) [n(%)] and were compared using Fisher’s exact test when the expected frequency of any cell was <5, otherwise the chi-square test was applied. Cramér’s V is reported as the effect size for categorical variables, and epsilon-squared (ϵ²) for continuous variables, both with 95% confidence intervals estimated via bootstrapping. All P-values were adjusted using the Benjamini–Hochberg procedure for False Discovery Rate (FDR) control and are reported as q-values. Given that all variables contributed to cluster construction, between-group differences are algorithmically inherent; thus, effect sizes and q-values are presented solely to quantify the magnitude of differences across subtype profiles, rather than for independent confirmatory inference.

#### 2.5.5 Sample size calculation

No universally accepted *a priori* sample size formula exists for exploratory cluster analysis (20). This study employed the Gower dissimilarity and PAM algorithm for subtype identification (21); PAM selects K based on internal indices such as the silhouette coefficient, Calinski-Harabasz (CH), and Davies-Bouldin (DB) indices and does not rely on power calculations per se. Sample adequacy was evaluated based on cluster size magnitude and resampling stability: the primary analysis included 500 independent patients, with a minimum cluster size of n=211 under K = 2. This far exceeds the reference lower bound of 20–30 cases per subgroup proposed by Dalmaijer et al. (which requires Δ≥4; our silhouette coefficient of 0.219 did not meet this threshold and serves only as a magnitude reference). The 1000 bootstrap iterations yielded a mean ARI of 0.916 (SD 0.073), and the within-cluster co-membership probability was 0.787, compared to 0.418 between clusters. Thus, 500 cases constitute a sufficient sample size for identifying K = 2 patient subtypes within the Gower-PAM framework.

## 3 Results

### 3.1 Descriptive statistics

Apr 7, 2025–Jan 21, 2026: 556 patients screened; 500 enrolled after excluding 38 ineligible and 18 refusing participation (effective response rate: 89.93%). Observation period: 905 AE occurrences recorded. To correct non-independence bias from multiple event reports per patient, the primary analysis randomly selected one event per patient ID, forming a 500-independent-sample dataset; the sensitivity analysis used the full 905-event dataset (**Figure 1**).

**Figure 1:**
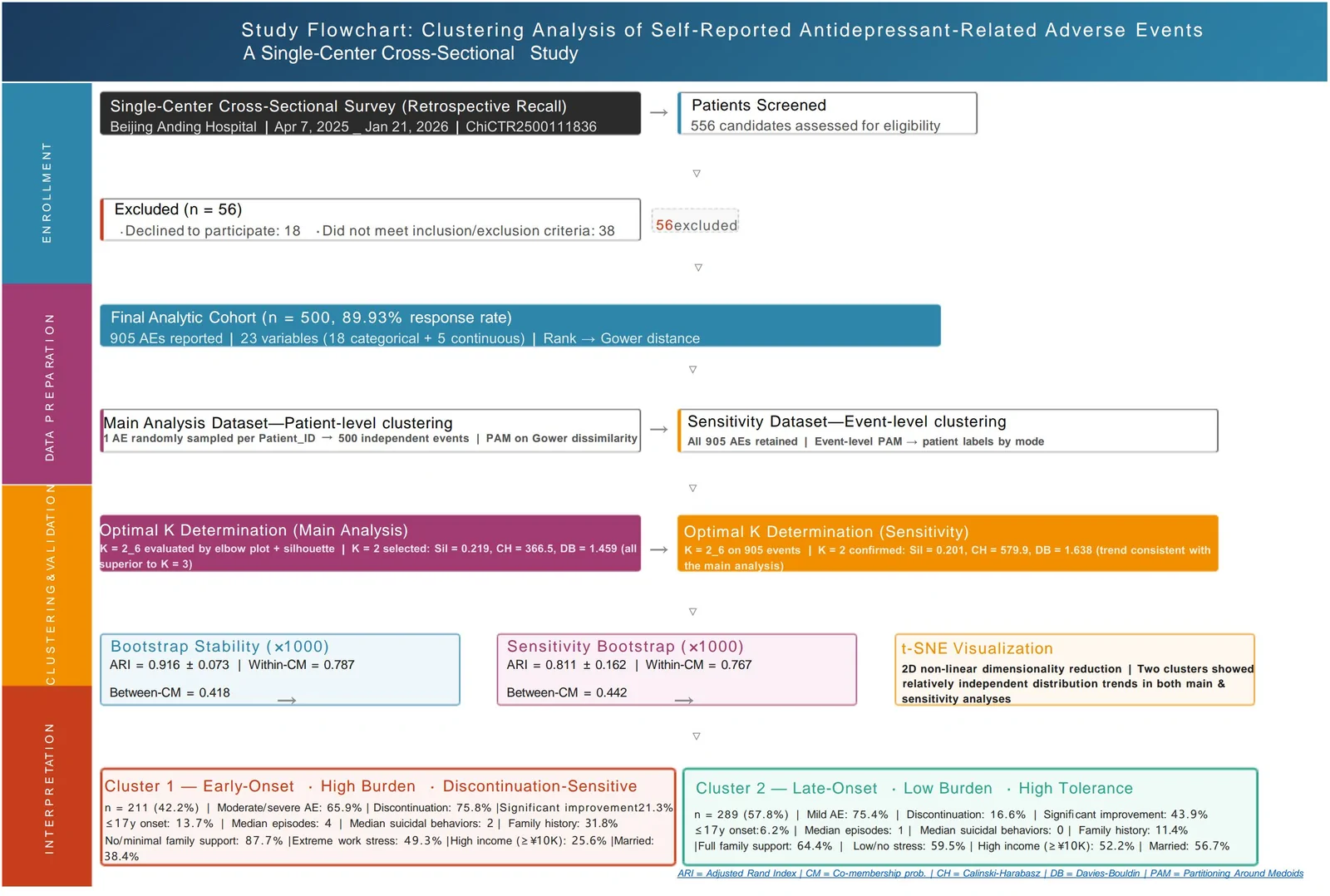
Study flowchart. Based on 500 patients (905 adverse event episodes), dual clustering models were constructed for the main analysis (patient-level) and sensitivity analysis (event-level). Bootstrap resampling confirmed the stability of the K-2 partition (ARI-0.916/0.811), identifying an Early-onset/High-burden/Discontinuation-sensitive subtype (42.2%) and a Late-onset/Low-burden/High-tolerant subtype (57.8%).

As shown in **Table 1**, females accounted for 67.4%; median age was 36 years (IQR 27–44). A total of 41.8% (209/500) had attained at least a middle school education. Median age at first onset was 31 years (IQR 23–40). Clinically, 36.2% (181/500) were first-episode, and 85.0% (425/500) were diagnosed with major depressive disorder. Over the past year, the number of antidepressant agents implicated in AEs per participant ranged from 1 to 3; the number of AEs induced by a single agent ranged from 1 to 4; and the cumulative number of AEs per participant ranged from 1 to 7. A total of 55.4% (277/500) of patients experienced at least two antidepressant-related AEs.

**Table 1:** Participant demographic and clinical characteristics.

Variables	Total	Variables	Total
	(n = 500)		(n = 500)
Age, median [IQR], year	36.0(27.0,44.0)	Insurance, No. (%)	
Sex, No. (%)		Publicly funded	72(14.4)
Male	163(32.6)	Cooperative medical care	14(2.8)
Female	337(67.4)	Basic medical insurance	318(63.6)
Educational level, No. (%)		Self-funded	96(19.2)
Elementary school or below	44(8.8)	Number of Past High - risk Factors No. (%)	
Middle school	55(11.0)	Zero	237(47.4)
Junior high school	110(22.0)	One	147(29.4)
Undergraduate school or higher	291(58.2)	Two	95(19.0)
Housing type, No. (%)		There	19(3.8)
Living alone	51(10.2)	Four times and more	2(0.4)
Living with strangers	9(1.8)	Primary Diagnosis, No. (%)	
Living with classmates, colleagues, or friends	60(12.0)	Depression	425(85.0)
Living with family	380(76.0)	Others^a^	75(15.0)
Patient family support, No. (%)		Age of onset, median [IQR], year	31.0(23.0,40.0)
No support	89(17.8)	Number of episodes, No. (%)	
Minimal support	128(25.6)	One	181(36.2)
Moderate support	94(18.8)	Two	116(23.2)
Full support	189(37.8)	There	75(15.0)
Marital status, No. (%)		Four times and more	128(25.6)
Divorced or widowed	37(7.4)	Comorbid other mental disorders, yes, No. (%)	285(57.0)
Unmarried	218(43.6)	One	269(53.8)
Married	245(49.0)	Two	15(3.0)
Work stress, No. (%)		Three kinds and more	1(0.2)
Extremely high	126(25.2)	Comorbid with somatic disease,yes, No. (%)	81(16.2)
High	107(21.4)	One	59(11.8)
Moderate	68(13.6)	Two	12(2.4)
Low	94(18.8)	Three kinds and more	10(2.0)
None	105(21.0)	Number of antidepressant with ADRs within the past year, No. (%)	
Monthly income per household member (RMB, Yuan), No. (%)		One	451(90.2)
1000 and below	6(1.2)	Two	48(9.6)
1000∼2999	28(5.6)	Three kinds and more	1(0.2)
3000∼4999	105(21.0)	Number of AEs within the past year for each patients, No. (%)	
5000∼9999	156(31.2)		
10000 and above	205(41.0)	One	223(44.6)
Regular drinking history, yes, No. (%)	23(4.6)	Two	172(34.4)
Smoking history, yes, No. (%)	67(13.4)	Three	91(18.2)
Family history of psychiatric disorders, yes, No. (%)	100(20.0)	Four and more^b^	14(2.8)

a 75 patients were diagnosed as other mental disorders: 14 diagnosed as schizophrenia;48 diagnosed as bipolar disorder;12 diagnosed as anxiety disorders; one diagnosed as feeding and eating disorders.

b 14 patients experienced three or more AEs due to antidepressant agents within the past year:9 experienced 4 AEs; 2 experienced 5 AEs; 2 experienced 6 AEs; 1 experienced 7 AEs.

### 3.2 Cluster analysis based on Gower distance and the PAM algorithm

#### 3.2.1 Determination of cluster number and result stability

To determine the optimal number of clusters, K = 2 and K = 3 schemes were compared. K = 2 outperformed K = 3 across all metrics: silhouette coefficient (0.219 vs. 0.088; **Figure 2**), CH (CH:366.510 vs. 177.880), and DB (DB:1.459 vs. 2.441). The K = 2 sample distribution (211/289) was more balanced than K = 3 (200/140/160), mitigating statistical volatility in smaller clusters during Bootstrap resampling. Integrating quantitative metrics and clinical interpretability, K = 2 was selected as optimal.

**Figure 2:**
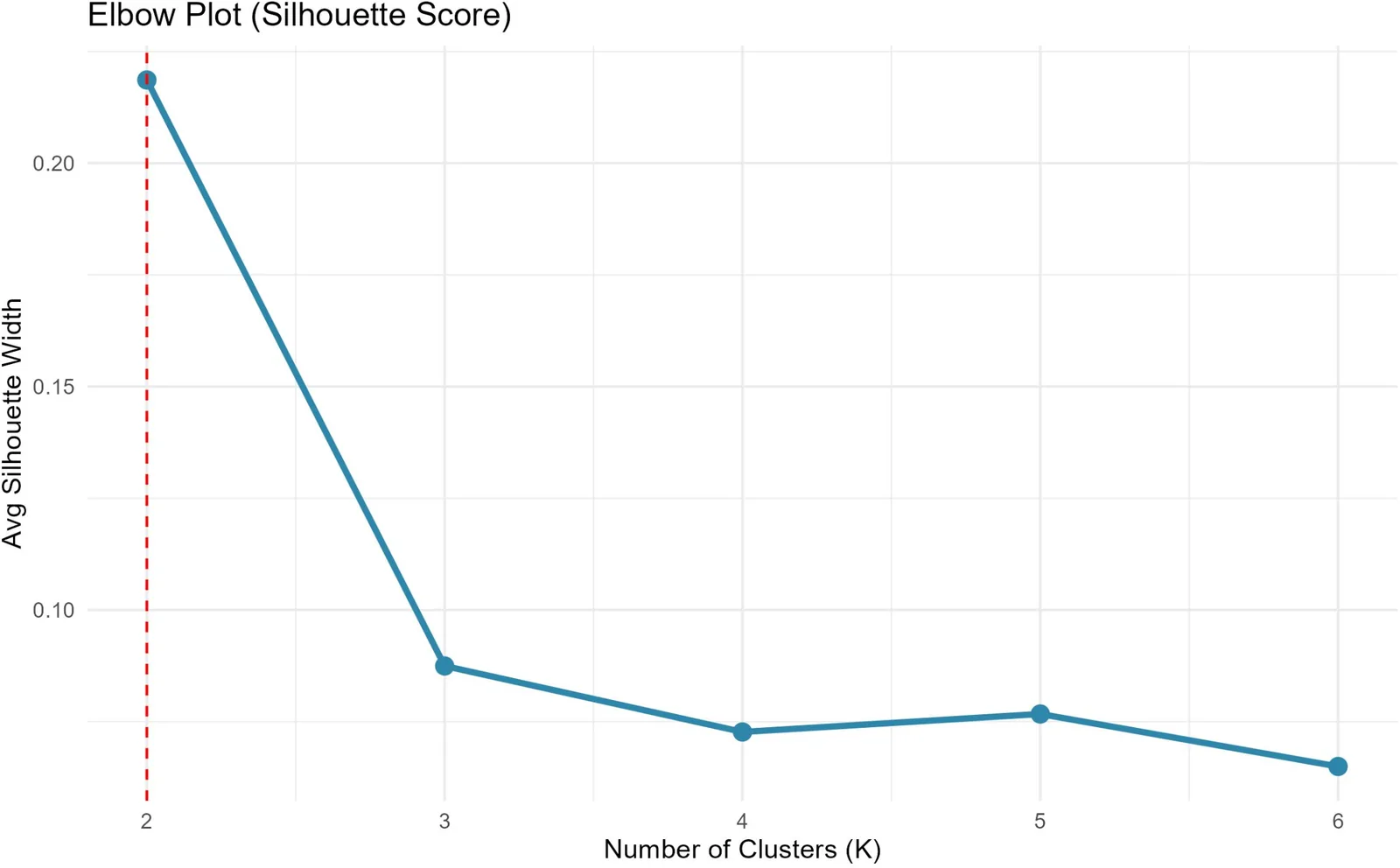
Elbow plot for selecting the optimal number of clusters (K) in the main analysis. The horizontal axis represents candidate cluster numbers (K=2-6), and the vertical axis represents the average silhouette width. The red dashed line indicates the selected optimal cluster number K-2 (coefficient≈0.219), which is substantially higher than that of K-3 (≈0.088) and higher K values. Although the absolute coefficient value is attenuated by the high heterogeneity of the study population and the use of Gower distance, the marked increase from K=3 to K-2 supports K-2 as the optimal cluster number.

Internal validation via 1000 Bootstrap resamplings supported K = 2 stability: mean ARI was 0.916 (SD = 0.073) (**Figure 3**) versus 0.509 (SD = 0.141) for K = 3, indicating high concordance with the reference solution. Overall co-membership probability was 0.607 (SD = 0.220); within-cluster probability (0.787) exceeded between-cluster (0.418) by ~0.37, consistent with a distinct cluster structure. The high, low-variance ARI and significant within-/between-cluster probability gap confirmed robust stability, not random error. t-SNE visualization (**Figure 4**) showed relatively distinct subgroup distributions; limited overlap aligned with inherent heterogeneity, supporting partition validity.

**Figure 3:**
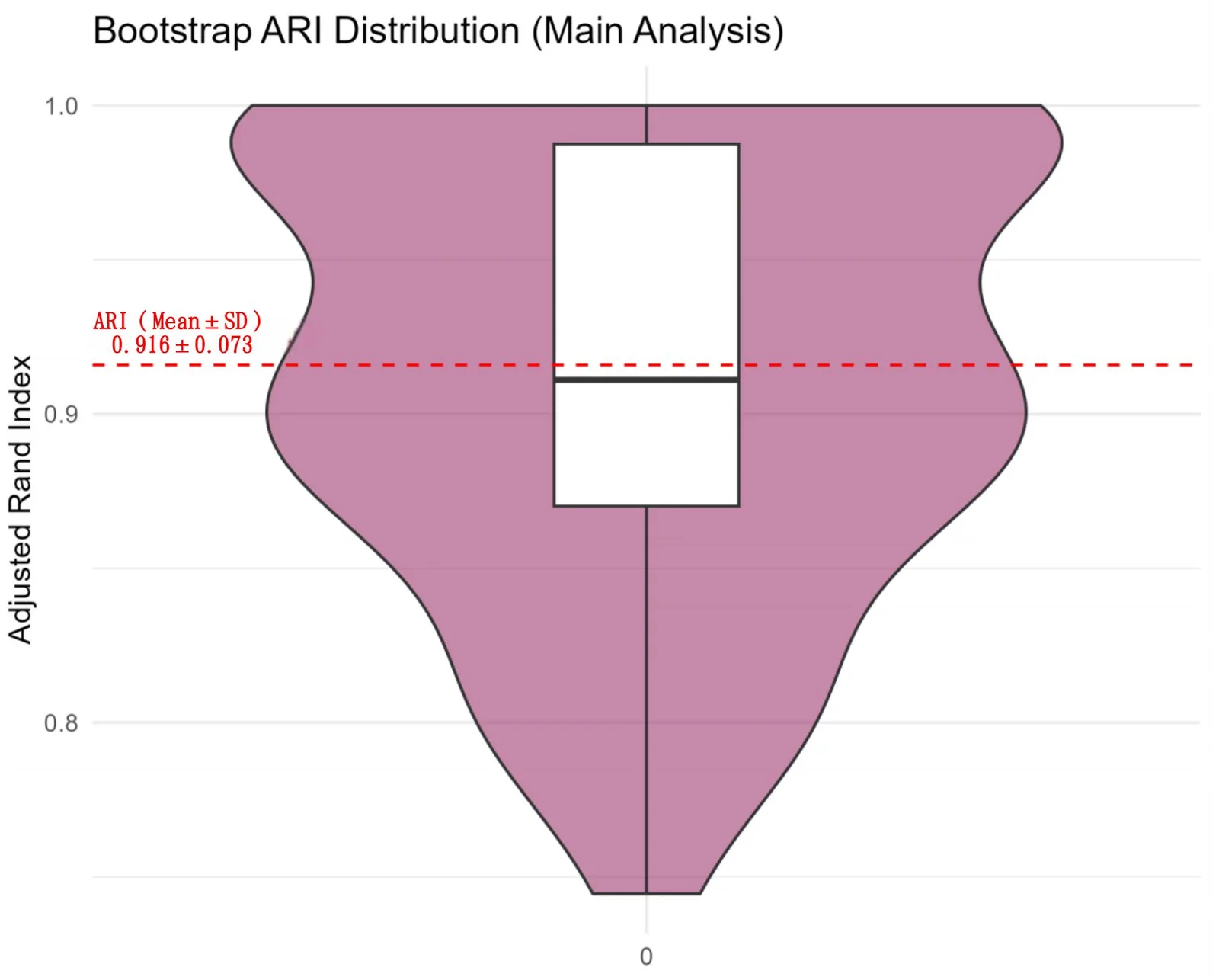
Distribution of the Adjusted Rand Index (ARI) from Bootstrap internal validation of the K-2 clustering solution in the main analysis. Stability was assessed using 1000 Bootstrap resamples, with the vertical axis representing ARI values. The red dashed line indicates the mean ± standard deviation (0.916+0.073). The overall distribution was concentrated within a high-value range, indicating relatively good stability of the K-2 clustering solution.

**Figure 4:**
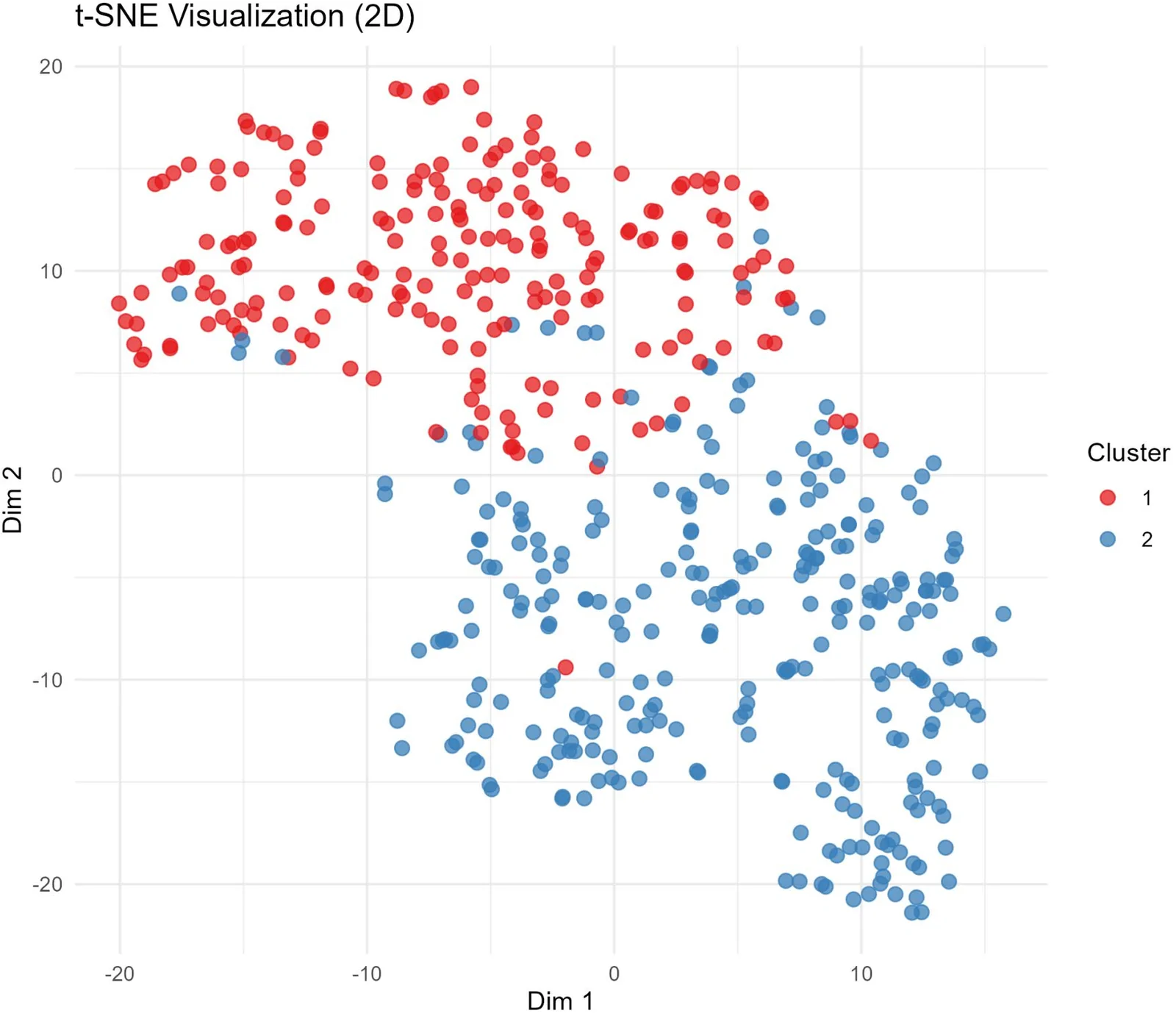
t-SNE visualization via non-linear dimensionality reduction of the K-2 clustering solution in the main analysis. In the two-dimensional scatter plot, the horizontal and vertical axes represent the first and second t-SNE dimensions (Dim 1 and Dim 2), respectively, with red and blue points denoting the two identified clusters. The results show a relatively independent distribution trend between the two clusters, although minor sample overlap was present (consistent with the inherent characteristics of highly heterogeneous cross-sectional data), further supporting the validity of the K-2 partition.

#### 3.2.2 Distribution of clinical characteristics across primary analysis clusters

Based on the K = 2 clustering model, the 500 patients were partitioned into Cluster 1 (n=211, 42.2%) and Cluster 2 (n=289, 57.8%). The distribution of characteristics across the two clusters is presented in **Table 2**. The between-group comparisons in **Table 2** are intended to characterize the feature profiles of distinct subtypes. As these variables were used for cluster construction, between-group differences are algorithmically inherent; thus, these comparisons are descriptive only and do not constitute independent external validation evidence.

**Table 2:** Clinical and demographic profiles of two distinct antidepressant-associated AEs subtype.

Variable	Cluster1	Cluster2	Overall	Effect Size (95% CI)	q value (FDR)	Test
Total number No. (%)	211(42.2%)	289(57.8%)	500(100%)			
Sex no. (%)			500 (100%)	V=0.037 (0.002–0.116)	0.408	Chi-square
Male	64 (30.3%)	99 (34.3%)	163 (32.6%)			
Female	147 (69.7%)	190 (65.7%)	337 (67.4%)			
Age no. (%)			500 (100%)	V=0.116 (0.028–0.204)	0.038	Chi-square
18-44y	149 (70.6%)	230 (79.6%)	379 (75.8%)			
45-59y	40 (19.0%)	32 (11.1%)	72 (14.4%)			
≥60y	22 (10.4%)	27 (9.3%)	49 (9.8%)			
Education_Level no. (%)			500 (100%)	V=0.188 (0.113–0.287)	<0.001	Chi-square
Elementary school or below	20 (9.5%)	24 (8.3%)	44 (8.8%)			
Middle school	32 (15.2%)	23 (8%)	55 (11.0%)			
Junior high school	58 (27.5%)	52 (18%)	110 (22.0%)			
Undergraduate school or higher	101 (47.9%)	190 (65.7%)	291 (58.2%)			
Housing_type no. (%)			500 (100%)	V=0.129 (0.067–0.224)	0.041	Fisher exact
Living alone	29 (13.7%)	22 (7.6%)	51 (10.2%)			
Living with strangers	6 (2.8%)	3 (1.0%)	9 (1.8%)			
Living with classmates, colleagues, or friends	27 (12.8%)	33 (11.4%)	60 (12.0%)			
Living with family	149 (70.6%)	231 (79.9%)	380 (76%)			
Family_Support no. (%)			500(100%)	V=0.783 (0.742–0.828)	<0.001	Fisher exact
No support	81 (38.4%)	8 (2.8%)	89 (17.8%)			
Minimal support	104 (49.3%)	24 (8.3%)	128 (25.6%)			
Moderate support	23 (10.9%)	71 (24.6%)	94 (18.8%)			
Full support	3 (1.4%)	186 (64.4%)	189 (37.8%)			
Marital_Status no. (%)			500 (100%)	V=0.199 (0.129–0.279)	<0.001	Chi-square
Divorced or widowed	24 (11.4%)	13 (4.5%)	37 (7.4%)			
Unmarried	106 (50.2%)	112 (38.8%)	218 (43.6%)			
Married	81 (38.4%)	164 (56.7%)	245 (49.0%)			
Work_Stress no. (%)			500 (100%)	V=0.585 (0.506–0.644)	<0.001	Chi-square
None	22 (10.4%)	83 (28.7%)	105 (21%)			
Low	5 (2.4%)	89 (30.8%)	94 (18.8%)			
Moderate	19 (9.0%)	49 (17.0%)	68 (13.6%)			
High	61 (28.9%)	46 (15.9%)	107 (21.4%)			
Extremely high	104 (49.3%)	22 (7.6%)	126 (25.2%)			
Monthly_income_per_household_member no. (%)			500 (100%)	V=0.283 (0.212–0.367)	<0.001	Fisher exact
1000 and below	3 (1.4%)	3 (1.0%)	6 (1.2%)			
1000∼2999	20 (9.5%)	8 (2.8%)	28 (5.6%)			
3000∼4999	53 (25.1%)	52 (18.0%)	105 (21.0%)			
5000∼9999	81 (38.4%)	75 (26.0%)	156 (31.2%)			
10000 and above	54 (25.6%)	151 (52.2%)	205 (41.0%)			
Medical_Insurance no. (%)			500 (100%)	V=0.207 (0.136–0.288)	<0.001	Chi-square
Publicly funded	30 (14.2%)	41 (14.2%)	71 (14.2%)			
Cooperative medical care	8 (3.8%)	6 (2.1%)	14 (2.8%)			
Basic medical insurance	114 (54.0%)	205 (70.9%)	319 (63.8%)			
Self-funded	59 (28.0%)	37 (12.8%)	96 (19.2%)			
Drinking_History no. (%)			500 (100%)	V=0.131 (0.040–0.226)	0.004	Chi-square
Drinking_History - No	194 (91.9%)	283 (97.9%)	477 (95.4%)			
Drinking_History – Yes	17 (8.1%)	6 (2.1%)	23 (4.6%)			
Smoking_History no. (%)			500 (100%)	V=0.157 (0.082–0.237)	<0.001	Chi-square
Smoking_History - No	169 (80.1%)	264 (91.3%)	433 (86.6%)			
Smoking_History – Yes	42 (19.9%)	25 (8.7%)	67 (13.4%)			
Family_history_of_psychiatric_disorders no. (%)			500 (100%)	V=0.246 (0.166–0.322)	<0.001	Chi-square
Family_history —No	144 (68.2%)	256 (88.6%)	400 (80.0%)			
Family_history —Yes	67 (31.8%)	33 (11.4%)	100 (20.0%)			
Number_of_Past_High_risk_Factors[M (Q1, Q3)]	2.0 [1.0, 2.0]	0.0 [0.0, 0.0]	1.0 [0.0, 1.0]	ϵ²=0.490 (0.430–0.572)	<0.001	Kruskal-Wallis
Number_of_Comorbid_Mental_Disorders[M(Q1,Q3)]	1.0 [1.0, 1.0]	0.0 [0.0, 1.0]	1.0 [0.0, 1.0]	ϵ²=0.457 (0.392–0.530)	<0.001	Kruskal-Wallis
Age_at_First_Onset_of_Disease no. (%)			500 (100%)	V=0.162 (0.093–0.249)	0.005	Chi-square
≤17y	29 (13.7%)	18 (6.2%)	47 (9.4%)			
18-44y	140 (66.4%)	221 (76.5%)	361 (72.2%)			
45-59y	35 (16.6%)	33 (11.4%)	68 (13.6%)			
≥60y	7 (3.3%)	17 (5.9%)	24 (4.8%)			
Number_of_episodes[M(Q1,Q3)]	4.0 [3.0, 5.0]	1.0 [1.0, 2.0]	2.0 [1.0, 4.0]	ϵ²=0.448 (0.380–0.516)	<0.001	Kruskal–Wallis
Number_of_Suicide_Behaviors_after_Diagnosis[M(Q1,Q3)]	2.0 [1.0, 4.0]	0.0 [0.0, 0.0]	0.0 [0.0, 2.0]	ϵ²=0.496 (0.410–0.580)	<0.001	Kruskal–Wallis
Number_of_Physical_Diseases[M (Q1, Q3)]	0.0 [0.0, 0.0]	0.0 [0.0, 0.0]	0.0 [0.0, 0.0]	ϵ²=0.027 (0.006–0.071)	<0.001	Kruskal–Wallis
Antidepressant_categeries no. (%)			500 (100%)	V=0.203 (0.160–0.298)	0.004	Fisher exact
SSRIs	109 (51.7%)	195 (67.5%)	304 (60.8%)			
SNRIs	42 (19.9%)	53 (18.3%)	95 (19.0%)			
NaSSA	7 (3.3%)	2 (0.7%)	9 (1.8%)			
SARI	26 (12.3%)	16 (5.5%)	42 (8.4%)			
Melatonin receptor agonists	5 (2.4%)	3 (1.0%)	8 (1.6%)			
Aminoketone antidepressants	8 (3.8%)	10 (3.5%)	18 (3.6%)			
Fixed-dose combination antidepressant	12 (5.7%)	9 (3.1%)	21 (4.2%)			
Multireceptor-acting antidepressant	2 (0.9%)	1 (0.3%)	3 (0.6%)			
Degree_of_Improvement_in_Depressive_Symptoms no. (%)			500 (100%)	V=0.261 (0.208–0.343)	<0.001	Fisher exact
Worse than before	2 (0.9%)	0 (0%)	2 (0.4%)			
No improvement	18 (8.5%)	8 (2.8%)	26 (5.2%)			
Mild improvement	56 (26.5%)	64 (22.1%)	120 (24.0%)			
Moderate improvement	90 (42.7%)	90 (31.1%)	180 (36.0%)			
Significant improvement	45 (21.3%)	127 (43.9%)	172 (34.4%)			
Different_organ_systems_of_AEs no. (%)			500 (100%)	V=0.237 (0.194–0.338)	0.002	Fisher exact
Digestive system	46 (21.8%)	108 (37.4%)	154 (30.8%)			
Cardiovascular system	32 (15.2%)	36 (12.5%)	68 (13.6%)			
Respiratory system	4 (1.9%)	2 (0.7%)	6 (1.2%)			
Nervous system	33 (15.6%)	40 (13.8%)	73 (14.6%)			
Reproductive system	12 (5.7%)	19 (6.6%)	31 (6.2%)			
Urinary system	3 (1.4%)	4 (1.4%)	7 (1.4%)			
Skin	1 (0.5%)	4 (1.4%)	5 (1.0%)			
Eye/ear/nose/throat	1 (0.5%)	6 (2.1%)	7 (1.4%)			
Hematologic system	5 (2.4%)	1 (0.3%)	6 (1.2%)			
Behavioral toxicity and sleep disorder	50 (23.7%)	37 (12.8%)	87 (17.4%)			
Metabolic system	19 (9.0%)	24 (8.3%)	43 (8.6%)			
Others^a^	5 (2.4%)	8 (2.8%)	13 (2.6%)			
Severity_of_AEs no. (%)			500 (100%)	V=0.439 (0.365–0.517)	<0.001	Fisher exact
Suspicious or mild	1 (0.5%)	6 (2.1%)	7 (1.4%)			
Mild	71 (33.6%)	218 (75.4%)	289 (57.8%)			
Moderate	109 (51.7%)	55 (19.0%)	164 (32.8%)			
Severe	30 (14.2%)	10 (3.5%)	40 (8.0%)			
Discontinue no. (%)			500 (100%)	V=0.589 (0.529–0.673)	<0.001	Chi-square
Discontinue – No	51 (24.2%)	241 (83.4%)	292 (58.4%)			
Discontinue - Yes	160 (75.8%)	48 (16.6%)	208 (41.6%)			

a Others include 3 psychomotor slowing,3 peripheral edema,2 asthenia with falls,2 restless-legs syndrome,1 derealization,1 formication and 1 pyrexia, data are derived from the "Other" category in **Supplementary Table S2** (Adverse Events reference table), encompassing adverse event types not included among the first 11 organ system categories, which patients self-list by name when completing **Supplementary Table S3** (Antidepressant Adverse Event Questionnaire). Antidepressant_categeries listed in **Table 2** are as follows: SSRIs (selective serotonin reuptake inhibitors): fluoxetine, sertraline, paroxetine, citalopram, fluvoxamine, escitalopram, and vortioxetine; SNRIs (serotonin and norepinephrine reuptake inhibitors): duloxetine, milnacipran, venlafaxine, and desvenlafaxine succinate; NaSSA (noradrenergic and specific serotonergic antidepressants): mirtazapine; aminoketone antidepressants: bupropion; melatonin receptor agonists: agomelatine; SARIs (serotonin antagonist and reuptake inhibitors): trazodone; fixed-dose combination antidepressants: flupentixol/melitracen combination and olanzapine/fluoxetine combination; multireceptor-acting antidepressants: tolvodesvenlafaxine. Effect sizes are reported as Cramér's V for categorical variables and epsilon-squared (ϵ²) for continuous variables, with 95% confidence intervals estimated via bootstrapping. q values represent significance levels after False Discovery Rate (FDR) correction using the Benjamini–Hochberg procedure. As all variables contributed to cluster construction, between-group differences are algorithmically inherent; thus, effect sizes and q values are presented solely to quantify the magnitude of differences across subtype profiles.

##### 3.2.2.1 Sociodemographic characteristics

Regarding demographic baselines, sex distribution showed no statistically significant difference (V = 0.037, 95% CI: 0.002–0.116, q=0.408), with both clusters predominantly female (Cluster 1: 69.7%; Cluster 2: 65.7%). Age distribution differed significantly (V = 0.116, 95% CI: 0.028–0.204, q=0.038), driven by a higher proportion of the 45–59 age group in Cluster 1 (19.0%) versus Cluster 2 (11.1%). Medical insurance type differed markedly (V = 0.207, 95% CI: 0.136–0.288, q<0.001), with a higher proportion of basic medical insurance in Cluster 2 (70.9%) than Cluster 1 (54.0%), while self-pay was more prevalent in Cluster 1 (28.0% vs. 12.8%).

For socioeconomic indicators, housing type differed significantly (q=0.041), with a higher rate of living alone in Cluster 1 (13.7% vs. 7.6%). Education level differed markedly (V = 0.188, 95% CI: 0.113–0.287, q<0.001), with a higher proportion of bachelor’s degree or above in Cluster 2 (65.7% vs. 47.9%). Monthly household income per capita also differed significantly (V = 0.283, 95% CI: 0.212–0.367, q<0.001), with a higher proportion of high-income individuals (≥10,000 CNY) in Cluster 2 (52.2% vs. 25.6%). Family support differed markedly (V = 0.783, 95% CI: 0.742–0.828, q<0.001), characterized by predominant full support in Cluster 2 (64.4%), whereas Cluster 1 was dominated by no support (38.4%) and minimal support (49.3%). Work stress differed substantially (V = 0.585, 95% CI: 0.506–0.644, q<0.001), with a higher proportion of extremely high stress in Cluster 1, while Cluster 2 was characterized by no stress (28.7%) and low stress (30.8%). Marital status also differed significantly (V = 0.199, 95% CI: 0.129–0.279, q<0.001), with a higher proportion of married individuals in Cluster 2 (56.7% vs. 38.4%).

For lifestyle and family history, drinking history differed significantly (V = 0.131, 95% CI: 0.040–0.226, q=0.004), with a higher positive rate in Cluster 1 (8.1% vs. 2.1%). Smoking history differed markedly (V = 0.157, 95% CI: 0.082–0.237, q<0.001), with a higher positive rate in Cluster 1 (19.9% vs. 8.7%). Family history of psychiatric disorders also differed significantly (V = 0.246, 95% CI: 0.166–0.322, q<0.001), with a higher positive rate in Cluster 1 (31.8% vs. 11.4%).

##### 3.2.2.2 Clinical severity, onset characteristics, and comorbidity

Age at onset differed significantly (V = 0.162, 95% CI: 0.093–0.249, q=0.005), with a higher proportion of onset at ≤17 years in Cluster 1 (13.7% vs. 6.2%). Number of physical diseases differed significantly (ϵ²=0.027, 95% CI: 0.006–0.071, q<0.001), with a median of 0.0 [0.0, 0.0] in both clusters, suggesting a difference in the distribution pattern of non-zero values despite an overall low burden. Number of suicide behaviors differed markedly (ϵ²=0.496, 95% CI: 0.410–0.580, q<0.001), with a higher median in Cluster 1 (2.0 [1.0, 4.0] vs. 0.0 [0.0, 0.0]). Number of episodes differed substantially (ϵ²=0.448, 95% CI: 0.380–0.516, q<0.001), with a higher median in Cluster 1 (4.0 [3.0, 5.0] vs. 1.0 [1.0, 2.0]). Number of past high-risk factors differed markedly (ϵ²=0.490, 95% CI: 0.430–0.572, q<0.001), with a higher median in Cluster 1 (2.0 [1.0, 2.0] vs. 0.0 [0.0, 0.0]). Number of comorbid mental disorders also differed significantly (ϵ²=0.457, 95% CI: 0.392–0.530, q<0.001), with a higher median in Cluster 1 (1.0 [1.0, 1.0] vs. 0.0 [0.0, 1.0]).

##### 3.2.2.3 Medication characteristics and AE profiles

Antidepressant category differed significantly (V = 0.203, 95% CI: 0.160–0.298, q=0.004), with selective serotonin reuptake inhibitors (SSRIs) predominating in Cluster 2 (67.5%). Systems involved in adverse events differed markedly (V = 0.237, 95% CI: 0.194–0.338, q=0.002), with a higher proportion of gastrointestinal events in Cluster 2 (37.4% vs. 21.8%), whereas behavioral toxicity and sleep disorders were more prevalent in Cluster 1 (23.7% vs. 12.8%). Discontinuation rates differed substantially (χ²=173.631, q<0.001), with a higher rate in Cluster 1 (75.8% vs. 16.6%). Severity of adverse drug reactions differed markedly (V = 0.439, 95% CI: 0.365–0.517, q<0.001), with a higher combined proportion of moderate (51.7%) and severe (14.2%) cases in Cluster 1 (65.9% vs. Cluster 2), whereas mild cases predominated in Cluster 2 (75.4%). Degree of depressive symptom improvement also differed significantly (V = 0.261, 95% CI: 0.208–0.343, q<0.001), with a higher proportion of marked improvement in Cluster 2 (43.9% vs. 21.3%).

##### 3.2.2.4 Overview of clustering results

Based on Gower distance and the PAM algorithm, 500 patients were partitioned into two clinically heterogeneous subtypes (**Table 2**):

Cluster 1 (n=211, 42.2%): Characterized by earlier onset (≤17 years: 13.7%), high disease burden (elevated high-risk factors, comorbidities, episodes, and suicidal behaviors), predominantly moderate-to-severe AEs (65.9%), and a high discontinuation rate (75.8%). This cluster exhibited poor social support (no/minimal support: 87.7%), high work stress (extremely high: 49.3%), higher rates of smoking and alcohol use, and a higher prevalence of family history of psychiatric disorders (31.8%), representing a high-risk clinical phenotype.

Cluster 2 (n=289, 57.8%): Characterized by later onset, low disease burden, predominantly mild AEs (75.4%), significant symptom improvement (43.9%), and a low discontinuation rate (16.6%). This cluster demonstrated strong social support (complete support: 64.4%), higher income (≥10,000 CNY: 52.2%), and predominant SSRI use (67.5%), representing a low-risk, high-tolerance phenotype.

### 3.3 Sensitivity analysis

#### 3.3.1 Validation of sensitivity analysis results

To assess robustness, sensitivity analysis used all 905 adverse event occurrences. For optimal cluster number, K = 2 outperformed K = 3 across metrics: silhouette 0.201 vs. 0.120; CH 579.940 vs. 334.720; DB 1.638 vs. 2.505 (**Supplementary Figure 1**). Although K = 2 silhouette was slightly lower than primary analysis (0.219), the relative trend supported a two-cluster structure. Bootstrap (1000 iterations) yielded K = 2 ARI 0.811 (SD = 0.162), within high concordance range (>0.8) (**Supplementary Figure 2**). Mean co-membership probability was 0.605 (SD = 0.207), Within-cluster probability (0.767) exceeded between-cluster (0.442), indicating stable intra-cluster co-occurrence and low cross-cluster consistency, consistent with distinct cluster structure. t-SNE showed relatively distinct subgroup distributions (**Supplementary Figure 3**). These findings confirm stable cluster structure aligning with primary analysis subtypes, supporting result reliability.

#### 3.3.2 Clinical characteristics of sensitivity analysis clusters

Sensitivity analysis of 905 events (PAM clustering + modal Patient_ID aggregation) partitioned 500 patients into Cluster 1 (n=232, 46.4%) and Cluster 2 (n=268, 53.6%), aligning with primary analysis (**Supplementary Table 5**).

Cluster 1 (46.4%):​ Earlier onset (≤17y: 13.8%); high burden (high-risk factors median:2.0; episodes median: 4.0; suicidal behaviors median: 2.0); poor support (no/minimal: 85.8%); high work stress (extreme: 47.4%); severe AEs (moderate/severe: 58.6%); high discontinuation (62.5%)—high-risk phenotype.

Cluster 2 (53.6%):​ Later onset; low burden (high-risk factors median: 0.0; episodes median: 1.0); family support (full: 68.3%); high income (≥10k CNY: 51.5%); predominant SSRI use (68.3%); mild AEs (77.6%); low discontinuation (5.6%); highest marked improvement (38.1%)—low-risk, high-tolerance phenotype.

## 4 Discussion

This study is an exploratory secondary analysis of data from a single-center, retrospective, recall-based cross-sectional survey. Using the Gower dissimilarity–based PAM algorithm, we performed exploratory cluster analysis on 500 patients who self-reported antidepressant-related AEs. Subtyping stability was evaluated via 1000 patient-level bootstrap resamples, using the ARI as the core metric. Results indicated that K = 2 was the optimal clustering solution, yielding a mean ARI of 0.912 (SD = 0.086). The all-event sensitivity analysis produced a mean ARI of 0.826, supporting the adequate stability of this two-cluster solution within our sample. However, the silhouette coefficient suggested a degree of phenotypic overlap between clusters, which aligns with the inherent characteristics of highly heterogeneous, self-report cross-sectional data. Consequently, the two identified categories do not represent disease subtypes with absolutely clear boundaries; rather, they reflect concomitant patterns linking AE burden, psychosocial resources, and tolerability. Cluster 1 (n = 211, 42.2%) was characterized by early onset, high disease burden, and a high discontinuation rate—reaching 75.8%, with moderate-to-severe AEs accounting for 65.9%, and only 21.3% of patients reporting significant improvement in depressive symptoms. In contrast, Cluster 2 (n = 289, 57.8%) exhibited relatively later onset, lower disease burden, and higher tolerability, with a discontinuation rate of merely 16.6% and mild AEs comprising 75.4%.

Discontinuation due to AEs is a common confounding factor affecting treatment maintenance in antidepressant therapy. The strikingly high discontinuation rate of 75.8% in Cluster 1 aligns with existing evidence on how antidepressant AEs compromise medication adherence. A retrospective cohort study reported that adverse events such as anxiety, agitation, suicidal ideation, and rash were associated with increased discontinuation risk, whereas sexual dysfunction and weight gain did not elevate this risk—indicating that not all AEs contribute equally to treatment discontinuation (5).

Our findings partly reflect this complexity. Regarding affected organ systems, Cluster 1 had a significantly higher proportion of behavioral toxicity and sleep disturbance AEs (23.7%) compared to Cluster 2 (12.8%), while Cluster 2 exhibited a higher prevalence of gastrointestinal AEs (37.4% vs. 21.8% in Cluster 1). Concurrently, moderate-to-severe AEs accounted for 65.9% of cases in Cluster 1, corresponding to its 75.8% discontinuation rate, whereas Cluster 2 was predominantly characterized by mild AEs (75.4%) and a markedly lower discontinuation rate of 16.6%. This concomitant pattern suggests that high discontinuation rates may be associated with specific AE phenotypes (e.g., behavioral toxicity, sleep disturbance) and AE severity, rather than merely the number of AEs or involvement of a common organ system such as the gastrointestinal tract.

Regarding antidepressant classes, Garcia-Marin et al. reported the highest discontinuation rates with mirtazapine and amitriptyline (57.3% and 51.6%, respectively) among 10 antidepressants (5). In our study, Cluster 2 was predominantly composed of SSRI users (67.5%), while Cluster 1 showed a relatively lower SSRI utilization (51.7%) but higher proportions of SARIs (12.3% vs. 5.5%), NaSSAs (3.3% vs. 0.7%), and fixed-dose combination antidepressants (5.7% vs. 3.1%). This specific “antidepressant class–AE phenotype” combination constitutes a pattern of treatment interruption, corroborating Garcia-Marin et al.’s findings that antidepressants with complex receptor profiles (e.g., mirtazapine, amitriptyline) are associated with higher discontinuation rates. This implies that the pharmacological properties of antidepressants may exacerbate discontinuation risk by shaping distinct AE profiles.

Associations between sociodemographic characteristics and AE tolerability. Real-world data from Karami et al. involving over 3.52 million patients and 8.92 million prescriptions in Iran demonstrated a sustained increase in antidepressant prescribing, with female patients accounting for 66.11% and prescription rates rising with age. Significant sex-based differences were also observed in the distribution of drug classes, including tricyclics, SNRIs, and atypical antidepressants (3). This evidence indicates heterogeneity in antidepressant exposure across sociodemographic subgroups, suggesting that AE tolerability may also vary in association with antidepressant class and psychosocial resources. Cluster 1 in our study was characterized by scarce psychosocial resources, as reflected by its combined profile: lacking family support (no support 38.4% + minimal support 49.3%), extremely high work stress (very high stress 49.3%), low income (monthly personal income <5000 CNY, 74.4%), high self-pay proportion (28.0%), and a high prevalence of being unmarried, divorced, or widowed (61.6%). This clustering of “low psychosocial resources—high AE severity—high discontinuation rate” suggests that, among patients who have already experienced AEs, psychosocial vulnerability may co-occur with low AE tolerability. Similarly, Read et al. reported that total AE scores were associated with lower educational attainment and lower income (4).

Associations between disease burden and AE phenotypes.​ Cluster 1 (high discontinuation) exhibited uniformly elevated disease burden: 13.7% onset ≤17 years; median episodes: 4; median suicidal behaviors: 2; median high-risk factors: 2; high comorbidity rates. This concomitant pattern suggests an association between greater disease burden and lower AE tolerability among patients with established AEs, potentially related to illness severity, treatment complexity, and overall health status. Such patients often require polypharmacy, increasing risks of drug interactions and AEs. Aarabi et al. reported potential drug interactions in 46.1% of >2.36 million outpatient prescriptions, with polypharmacy as a strong predictor (22). Retrospective cohort data linked antipsychotic polypharmacy to higher AE risk (23). In depressive disorders, severe/r recurrent illness correlates with antidepressant polypharmacy, where greater comorbidity counts amplify risks of interactions and inappropriate prescribing (24). Collectively, these findings indicate an association between higher disease burden/comorbidity and increased susceptibility to AEs, largely attributable to polypharmacy-related risks.

In summary, this single-center, retrospectively recalled cross-sectional study identified two patient subgroups with self-reported antidepressant-related AEs using Gower dissimilarity and PAM clustering—each exhibiting within-group homogeneity and between-group heterogeneity. Cluster 1 was characterized by high disease burden, moderate-to-severe AEs, high discontinuation rates, and limited psychosocial resources. Cluster 2 exhibited later onset, low disease burden, predominantly mild AEs, low discontinuation rates, and relatively sufficient psychosocial resources. As an exploratory pattern recognition study, these findings provide a hypothesis-generating basis for future prospective cohorts and external validation.

## 5 Limitations

This study has several limitations. First, as a single-center, retrospectively recalled cross-sectional study of patients with self-reported antidepressant-related AEs, findings apply only to this specific population and cannot be generalized to all antidepressant users or used to identify predictors of AE occurrence. Second, the unavailability of key data—including drug dosage, treatment duration, concomitant medications, and drug interactions—constitutes a major limitation of this study. Third, reliance on retrospective self-reports introduced recall bias. Symptoms (e.g., insomnia, fatigue, sexual dysfunction, attentional deficits) may relate to the underlying depressive disorder, comorbidities, or concomitant medications, as formal causality assessment using the Naranjo scale, WHO-UMC criteria, or clinician adjudication was not performed. Fourth, although Bootstrap validation supported the classification, the K = 2 silhouette coefficient (0.219) indicated partial cluster overlap, and cluster number selection was data-dependent. Fifth, the cross-sectional design precluded establishing temporal relationships between AE severity, psychosocial characteristics, and discontinuation behavior; reverse causality remains possible.

## 6 Future research directions

Building on these findings and limitations, future studies should: (1) Validate the identified subgroups in independent, multicenter, prospective cohorts and evaluate their predictive value for clinical outcomes. (2) Integrate pharmacokinetic and clinical pharmacy data (dosage, serum concentrations, treatment duration, concomitant medications, drug interactions) with causality assessment frameworks (e.g., Naranjo scale, WHO-UMC criteria) to enhance AE ascertainment. (3) Explore integrating such risk-stratification models into AI-enabled Clinical Decision Support Systems (CDSS) (25). This requires addressing ethical imperatives: the WHO mandates transparency, accountability, data privacy, and fairness in AI health applications (26); the Khosravi et al. AI Ethics Compliance Scale provides a measurable tool for assessing CDSS alignment with these principles (27). (4) Develop targeted interventions—e.g., individualized pharmaceutical care combined with psychosocial support—for high-risk subgroups (high burden, limited psychosocial resources) to improve adherence and clinical outcomes.

## Data Availability

The raw data supporting the conclusions of this article will be made available by the authors, without undue reservation.
