# Supplementary material for: Exploratory cluster analysis of self-reported adverse events associated with antidepressants using Gower distance and partitioning around medoids: a single-center cross-sectional study
Source: Front Psychiatry. 2026 Sep 17;17:1916987. doi: 10.3389/fpsyt.2026.1916987 (PMC13628165; doi:10.3389/fpsyt.2026.1916987)
Supplement: Supplementary file 1 [file Table1.docx]

**Table S1 |** **STROBE Statement Checklist for Cross-Sectional Studies, with Reference to Applicable Items from the RECORD Extension (for Routinely Collected Data)**

|  | Item No | Recommendation | Location in manuscript |
| --- | --- | --- | --- |
| **Title and abstract** | 1 | (*a*) Indicate the study’s design with a commonly used term in the title or the abstract | Abstract（page1） |
|  |  | (*b*) Provide in the abstract an informative and balanced summary of what was done and what was found | Abstract（page1） |
| Introduction | | |  |
| Background/rationale | 2 | Explain the scientific background and rationale for the investigation being reported | Research Background  (page2) |
| Objectives | 3 | State specific objectives, including any prespecified hypotheses | Research Background  (page2) |
| Methods | | |  |
| Study design | 4 | Present key elements of study design early in the paper | Study design(page3) Described as an exploratory secondary analysis of a single-center, retrospective-recall cross-sectional survey on antidepressant-related adverse events, registered in ChiCTR (ChiCTR2500111836) on November 6, 2025. |
| Setting | 5 | Describe the setting, locations, and relevant dates, including periods of recruitment, exposure, follow-up, and data collection | Study design(page3) |
| Participants | 6 | (*a*) Give the eligibility criteria, and the sources and methods of selection of participants | Participants(page3) |
| Variables | 7 | Clearly define all outcomes, exposures, predictors, potential confounders, and effect modifiers. Give diagnostic criteria, if applicable | Outcome measures  (page5) |
| Data sources/ measurement | 8* | For each variable of interest, give sources of data and details of methods of assessment (measurement). Describe comparability of assessment methods if there is more than one group | Outcome measures  (page5) Detailed the self-designed questionnaire as the data source. Addressed potential recall bias by restricting to adverse events occurring within the past 1 year and conducting sensitivity analyses. |
| Bias | 9 | Describe any efforts to address potential sources of bias | Limitations and Participants(page3,12,13) |
| Study size | 10 | Explain how the study size was arrived at | Statistical analysis  (page6) The sample size (n=500) was determined by the original survey for descriptive aims; adequacy for clustering was supported by Bootstrap stability (ARI=0.916). |
| Quantitative variables | 11 | Explain how quantitative variables were handled in the analyses. If applicable, describe which groupings were chosen and why | Statistical analysis  (page6) |
| Statistical methods | 12 | (*a*) Describe all statistical methods, including those used to control for confounding | Statistical analysis  (page6) Clarified that the 500 patients were consecutively enrolled from the outpatient clinic of Beijing Anding Hospital, representing a complete case series of the original survey rather than a random sample. |
|  |  | (*b*) Describe any methods used to examine subgroups and interactions |  |
|  |  | (*c*) Explain how missing data were addressed |  |
|  |  | (*d*) If applicable, describe analytical methods taking account of sampling strategy |  |
|  |  | (*e*) Describe any sensitivity analyses |  |
| Results | | |  |
| Participants | 13* | (a) Report numbers of individuals at each stage of study—eg numbers potentially eligible, examined for eligibility, confirmed eligible, included in the study, completing follow-up, and analysed | Descriptive statistics(page 7-8) |
|  |  | (b) Give reasons for non-participation at each stage |  |
|  |  | (c) Consider use of a flow diagram |  |
| Descriptive data | 14* | (a) Give characteristics of study participants (eg demographic, clinical, social) and information on exposures and potential confounders | Descriptive statistics(page 7-8) |
|  |  | (b) Indicate number of participants with missing data for each variable of interest |  |
| Outcome data | 15* | Report numbers of outcome events or summary measures | Cluster Results (page 8-11) |
| Main results | 16 | (*a*) Give unadjusted estimates and, if applicable, confounder-adjusted estimates and their precision (eg, 95% confidence interval). Make clear which confounders were adjusted for and why they were included | Cluster Results (page 8-11) |
|  |  | (*b*) Report category boundaries when continuous variables were categorized |  |
|  |  | (*c*) If relevant, consider translating estimates of relative risk into absolute risk for a meaningful time period |  |
| Other analyses | 17 | Report other analyses done—eg analyses of subgroups and interactions, and sensitivity analyses | sensitivity analyses  (page 10-11) |
| Discussion | | |  |
| Key results | 18 | Summarise key results with reference to study objectives | Discussion(page11-12) |
| Limitations | 19 | Discuss limitations of the study, taking into account sources of potential bias or imprecision. Discuss both direction and magnitude of any potential bias | Limitations (page12-13) |
| Interpretation | 20 | Give a cautious overall interpretation of results considering objectives, limitations, multiplicity of analyses, results from similar studies, and other relevant evidence | Limitations (page12-13) |
| Generalisability | 21 | Discuss the generalisability (external validity) of the study results | Discussion(page11-12) |
| Other information | | |  |
| Funding | 22 | Give the source of funding and the role of the funders for the present study and, if applicable, for the original study on which the present article is based | Acknowledgements(13) |
