# Supplementary material for: Exploratory cluster analysis of self-reported adverse events associated with antidepressants using Gower distance and partitioning around medoids: a single-center cross-sectional study
Source: Front Psychiatry. 2026 Sep 17;17:1916987. doi: 10.3389/fpsyt.2026.1916987 (PMC13628165; doi:10.3389/fpsyt.2026.1916987)
Supplement: Supplementary file 2 [file Table2.docx]

**Table S2| Adverse Event Reference Table**

| **Involved System** | **Adverse Events** |
| --- | --- |
| Digestive System | 1. Diarrhea; 2. Constipation; 3. Nausea/Vomiting; 4. Xerostomia; 5. Decreased Appetite |
| Cardiovascular System | 6. Palpitations; 7. Dizziness upon standing; 8. Chest Pain; 9. Hypertension; 10. Hypotension; 11. ECG Abnormalities; 12. Elevated Myocardial Enzymes |
| Respiratory System | 13. Shortness of Breath and Chest Tightness |
| Nervous System | 14. Headache; 15. Vertigo; 16. Tremor; 17. Rigidity; 18. Akathisia; 19. Hyperhidrosis; 20. Drooling (Excessive Salivation) |
| Reproductive System | 21. Reduced Sexual Function; 22. Menstrual Irregularity |
| Urinary System | 23. Dysuria; 24. Urinary frequency; 25. Urethritis; 26. Renal Dysfunction |
| Skin | 27. Rash; 28. Itching; 29. Purpura (Subcutaneous Hemorrhage Points) |
| Eye/Ear/Nose/Throat | 30. Blurred Vision; 31. Tinnitus; 32. Nasal Congestion; |
| Hematologic System | 33. Leukopenia; 34. Anemia; 35. Thrombocytopenia |
| Behavioral Toxicity and Sleep disorder | 36. Disturbance of Consciousness; 37. Agitation and Excitement; 38. Increase or Decrease in Motor Activity; 39. Insomnia; 40. Somnolence; 41. Sleep Quality Deterioration |
| Metabolic System | 42. Lipid Abnormalities; 43. Liver Function Abnormalities (Elevated Transaminases); 44. Weight Gain/Loss |
| Others | 45. Edema; 46. Other: Please specify in the "Adverse Reaction Survey Form for Antidepressants" |

**Note:** This *Adverse Event Reference Table* serves as a terminology aid tool to help you accurately report the names of adverse events (AEs) in the *Antidepressant Adverse Event Questionnaire*. If an AE you experience is not listed in this table, please simply specify the exact AE you experienced directly in the *Antidepressant Adverse Event Questionnaire*.
