# Supplementary material for: Exploratory cluster analysis of self-reported adverse events associated with antidepressants using Gower distance and partitioning around medoids: a single-center cross-sectional study
Source: Front Psychiatry. 2026 Sep 17;17:1916987. doi: 10.3389/fpsyt.2026.1916987 (PMC13628165; doi:10.3389/fpsyt.2026.1916987)
Supplement: Supplementary file 3 [file Table3.docx]

**Table S3|** **Antidepressant Adverse Event Questionnaire**

| **Antidepressant Type** | **Antidepressant 1:** |
| --- | --- |
| Adverse AE Types (Refer to *Adverse Event Reference Table*) | Adverse AE 1： |
| Dosage and Administration Method | Daily： times, each time： mg |
| Whether Medication Was Taken as Prescribed | □Yes □No |
| Depression Symptom Improvement (Based on Self-Perception) | □Significant Improvement (Essentially Recovered) □Moderate Improvement (Recovered Over 50%) □Mild Improvement (Improvement <50%) □No Improvement □Worse Than Before |
| Whether AEs Occurred | □Yes □No |
| Time of AE Onset | □Within 2 Weeks of Taking the Dose □2 Weeks to 3 Months After Taking the Dose □3 to 6 Months After Taking the Dose □More Than 6 Months After Taking the Dose |
| Severity of AE | □Suspicious or Mild □Mild (Tolerable, No Impact) □Moderate (Intolerable, Affects Normal Life/Work/Study) □Severe (Extremely Painful, Severely Affects Health and Normal Life/Work/Study) |
| Whether AE Relieved Spontaneously | □Yes □No |
| Management Plan for AE | □Increased Observation □Drug Dosage Reduction □Antagonist Controlled □Dosage Reduction and Antagonist Controlled □Discontinuation of the Drug |
| Whether AE Persisted After Intervention | □Yes □No |

Note: 1.For types of adverse events (AEs), please refer to the *Adverse Event Reference Table* and record the corresponding AE number (1, 2, 3, etc.). Any ADR not listed in the Adverse Event Reference Table should be described in this table directly.2.Criteria for Determining Depression Symptom Improvement: The improvement referred to herein denotes the patient's self-perceived change in depressive symptoms at the time of the adverse event occurrence, rather than representing the final assessment over the entire treatment cycle. The criteria are: Significant improvement (basically recovered); Moderate improvement (symptom relief ≥50%); Mild improvement (symptom relief <50%); No improvement (no change compared to pre-treatment status); Worsened (depressive symptoms worsened compared to pre-treatment status).3.Grading Criteria for Adverse Event Severity: Suspected or mild (suspected reaction with no obvious discomfort); Mild (tolerable, not affecting normal life/work/studies); Moderate (intolerable, affecting normal life/work/studies); Severe (extreme distress, severely impacting physical and mental health as well as normal life/work/studies).4. The Time of AE Onset is defined as the time interval self-reported by the patient from the initiation of antidepressant medication to the first onset of AE symptoms.5. Discontinuation is defined as the patient-initiated or clinician-advised cessation of antidepressant use specifically due to the reported adverse event.
