# Supplementary material for: Exploratory cluster analysis of self-reported adverse events associated with antidepressants using Gower distance and partitioning around medoids: a single-center cross-sectional study
Source: Front Psychiatry. 2026 Sep 17;17:1916987. doi: 10.3389/fpsyt.2026.1916987 (PMC13628165; doi:10.3389/fpsyt.2026.1916987)
Supplement: Supplementary file 4 [file Table4.docx]

Table S4**|** Variable Preprocessing and Contribution to Gower Distance for Cluster Analysis

| Variable | Type | Encoding | Included_in_Gower | Gower_Weight | Merged_Levels | Missing_N | Contribution_to_Distance |
| --- | --- | --- | --- | --- | --- | --- | --- |
| Antidepressant_categories | Categorical | Factor (levels with n<5 merged into 'Other') | Yes | 1 | Antidepressant_categories - 7 | 0 | 1.7382 |
| Different_organ_systems_of_ADR | Categorical | Factor (no merging) | Yes | 1 | None | 0 | 2.839 |
| Severity_of_ADRs | Categorical | Factor (no merging) | Yes | 1 | None | 0 | 1.3441 |
| Degree_of_Improvement_in_Depressive_Symptoms | Categorical | Factor (levels with n<5 merged into 'Other') | Yes | 1 | Degree_of_Improvement_in_Depressive_Symptoms - 1 | 0 | 1.8354 |
| Discontinue | Categorical | Factor (no merging) | Yes | 1 | None | 0 | 0.9619 |
| Gender | Categorical | Factor (no merging) | Yes | 1 | None | 0 | 0.8846 |
| age | Categorical | Factor (no merging) | Yes | 1 | None | 0 | 0.9569 |
| Age_at_First_Onset_of_Disease | Categorical | Factor (no merging) | Yes | 1 | None | 0 | 1.1957 |
| Education_Level | Categorical | Factor (no merging) | Yes | 1 | None | 0 | 1.5156 |
| Housing_type | Categorical | Factor (no merging) | Yes | 1 | None | 0 | 1.2005 |
| Family_Support | Categorical | Factor (no merging) | Yes | 1 | None | 0 | 1.9524 |
| Marital_Status | Categorical | Factor (no merging) | Yes | 1 | None | 0 | 1.3126 |
| Work_Stress | Categorical | Factor (no merging) | Yes | 1 | None | 0 | 2.2915 |
| Monthly_income_per_household_member | Categorical | Factor (no merging) | Yes | 1 | None | 0 | 1.8656 |
| Medical_Insurance | Categorical | Factor (no merging) | Yes | 1 | None | 0 | 1.3981 |
| Drinking_History | Categorical | Factor (no merging) | Yes | 1 | None | 0 | 0.2992 |
| Smoking_History | Categorical | Factor (no merging) | Yes | 1 | None | 0 | 0.5937 |
| Family_history_of_psychiatric_disorders | Categorical | Factor (no merging) | Yes | 1 | None | 0 | 0.766 |
| Number_of_Past_High_risk_Factors | Continuous | Rank-transformed (average tie handling) | Yes | 1 | NA | 0 | 1.0637 |
| Number_of_Comorbid_Mental_Disorders | Continuous | Rank-transformed (average tie handling) | Yes | 1 | NA | 0 | 0.8975 |
| Number_of_episodes | Continuous | Rank-transformed (average tie handling) | Yes | 1 | NA | 0 | 0.7326 |
| Number_of_Suicide_Behaviors_after_Diagnosis | Continuous | Rank-transformed (average tie handling) | Yes | 1 | NA | 0 | 1.8022 |
| Number_of_Physical_Diseases | Continuous | Rank-transformed (average tie handling) | Yes | 1 | NA | 0 | 2.7956 |

**Note:**1.**Merging Rules:** Levels with frequencies less than 5 in categorical variables were collapsed into an "Other" category. Specifically: ① For antidepressant categories, Level 7 ("Multireceptor-acting antidepressant," n=3) was merged into the Other group. As all other levels within this variable exceeded 5 cases, the Other group comprised only this single category. ② For the degree of improvement in depressive symptoms, Level 1 ("Worse than before," n=2) was merged into the Other group. As all other levels within this variable exceeded 5 cases, the Other group comprised only this single category.2.**Coding Rules:** Specification of variable coding :Sex assigned 1 for male, 2 for female; Education Level assigned 1 for elementary school or below,2 for middle school,3 for junior high school,4 for undergraduate school or higher; housing type assigned 1 for living alone,2 for living with strangers,3 for living with classmates, colleagues, or friends,4 for living with family; Family Support assigned 1 for no support,2 for minimal support ,3 for moderate support,4 for full support; Marital Status assigned 1 for divorced or widowed,2 for unmarried,3 for married; Work Stress assigned 1 for none,2 for low ;3 for moderate,4 for high,5 for extremely high; Monthly income per household member assigned 1 for 1000 and below,2 for 1000～2999,3 for 3000～4999,4 for 5000～9999,5 for 10000 and above; Medical Insurance assigned 1 for publicly funded,2 for cooperative medical care,3 for basic medical insurance,4 for self-funded; Drinking History assigned 1 for no, 2 for yes; Smoking History assigned 1 for no, 2 for yes; Family history of psychiatric disorders assigned1 for no, 2 for yes; Diagnosis assigned 1 for depression,2 for anxiety disorders,3 for bipolar disorder,4 for schizophrenia,5 for feeding and eating disorders; Degree of Improvement in Depressive Symptoms assigned 1 for worse than before,2 for no improvement,3 for mild improvement,4 for moderate improvement,5 for significant improvement; Antidepressant categories assigned 1 for ssris,2 for snris,3 for nassa,4 for saris,5 for melatonin receptor agonists,6 for aminoketone antidepressants,7 for multireceptor-acting antidepressant and 8 for fixed-dose combination antidepressant; Different organ systems assigned 1 for digestive system,2 for cardiovascular system,3 for respiratory system,4 for nervous system,5 for reproductive system,6 for urinary system,7 for skin, 8 for eye/ear/nose/throat, 9 for hematologic system,10 for behavioral toxicity and sleep disorder,11 for metabolic system, and 12 for others; Severity of ADRs assigned 1 for suspicious or mild,2 for mild,3 for moderate,4 for severe. Age and Age at First Onset of Disease assigned 1 for≤17,2 for 18 to 44,3 for 45 to 59,4 for≥60.
