# Supplementary material for: Exploratory cluster analysis of self-reported adverse events associated with antidepressants using Gower distance and partitioning around medoids: a single-center cross-sectional study
Source: Front Psychiatry. 2026 Sep 17;17:1916987. doi: 10.3389/fpsyt.2026.1916987 (PMC13628165; doi:10.3389/fpsyt.2026.1916987)
Supplement: Supplementary file 5 [file Table5.docx]

**Table S5 | Clinical and demographic profiles of two antidepressant-associated AEs subtypes in the sensitivity analysis**

| Variable | Cluster1 | Cluster2 | Overall | Effect Size  (95% CI) | q value (FDR) | Test |
| --- | --- | --- | --- | --- | --- | --- |
| Total number No. (%) | 232(46.4%) | 268(53.6%) | 500(100%) |  |  |  |
| Sex No. (%) |  |  | 500 (100%) | V=0.035 (0.002–0.117) | 0.429 | Chi-square |
| Male | 71 (30.6%) | 92 (34.3%) | 163 (32.6%) |  |  |  |
| Female | 161 (69.4%) | 176 (65.7%) | 337 (67.4%) |  |  |  |
| age No. (%) |  |  | 500 (100%) | V=0.164 (0.086–0.258) | 0.001 | Chi-square |
| 18-44y | 160 (69.0%) | 219 (81.7%) | 379 (75.8%) |  |  |  |
| 45-59y | 47 (20.3%) | 25 (9.3%) | 72 (14.4%) |  |  |  |
| ≥60y | 25 (10.8%) | 24 (9.0%) | 49 (9.8%) |  |  |  |
| Education_Level No. (%) |  |  | 500 (100%) | V=0.23 (0.160–0.308) | <0.001 | Chi-square |
| Elementary school or below | 25 (10.8%) | 19 (7.1%) | 44 (8.8%) |  |  |  |
| Middle school | 34 (14.7%) | 21 (7.8%) | 55 (11.0%) |  |  |  |
| Junior high school | 66 (28.4%) | 44 (16.4%) | 110 (22.0%) |  |  |  |
| Undergraduate school or higher | 107 (46.1%) | 184 (68.7%) | 291 (58.2%) |  |  |  |
| Housing_type No. (%) |  |  | 500 (100%) | V=0.129 (0.067–0.218) | 0.037 | Fisher exact |
| Living alone | 33 (14.2%) | 18 (6.7%) | 51 (10.2%) |  |  |  |
| Living with strangers | 5 (2.2%) | 4 (1.5%) | 9 (1.8%) |  |  |  |
| Living with classmates, colleagues, or friends | 28 (12.1%) | 32 (11.9%) | 60 (12.0%) |  |  |  |
| Living with family | 166 (71.6%) | 214 (79.9%) | 380 (76.0%) |  |  |  |
| Family_Support No. (%) |  |  | 500(100%) | V=0.816 (0.767–0.856) | <0.001 | Chi-square |
| No support | 83 (35.8%) | 6 (2.2%) | 89 (17.8%) |  |  |  |
| Minimal support | 116 (50.0%) | 12 (4.5%) | 128 (25.6%) |  |  |  |
| Moderate support | 27 (11.6%) | 67 (25.0%) | 94 (18.8%) |  |  |  |
| Full support | 6 (2.6%) | 183 (68.3%) | 189 (37.8%) |  |  |  |
| Marital_Status No. (%) |  |  | 500 (100%) | V=0.184 (0.103–0.273) | <0.001 | Chi-square |
| Divorced or widowed | 26 (11.2%) | 11 (4.1%) | 37 (7.4%) |  |  |  |
| Unmarried | 112 (48.3%) | 106 (39.6%) | 218 (43.6%) |  |  |  |
| Married | 94 (40.5%) | 151 (56.3%) | 245 (49.0%) |  |  |  |
| Work_Stress No. (%) |  |  | 500 (100%) | V=0.617 (0.555–0.684) | <0.001 | Chi-square |
| None | 24 (10.3%) | 81 (30.2%) | 105 (21.0%) |  |  |  |
| Low | 7 (3.0%) | 87 (32.5%) | 94 (18.8%) |  |  |  |
| Moderate | 20 (8.6%) | 48 (17.9%) | 68 (13.6%) |  |  |  |
| High | 71 (30.6%) | 36 (13.4%) | 107 (21.4%) |  |  |  |
| Extremely high | 110 (47.4%) | 16 (6.0%) | 126 (25.2%) |  |  |  |
| Monthly_income_per_household_member No. (%) | |  | 500 (100%) | V=0.243 (0.169–0.317) | <0.001 | Fisher exact |
| 1000 and below | 3 (1.3%) | 3 (1.1%) | 6 (1.2%) |  |  |  |
| 1000～2999 | 20 (8.6%) | 8 (3.0%) | 28 (5.6%) |  |  |  |
| 3000～4999 | 56 (24.1%) | 49 (18.3%) | 105 (21.0%) |  |  |  |
| 5000～9999 | 86 (37.1%) | 70 (26.1%) | 156 (31.2%) |  |  |  |
| 10000 and above | 67 (28.9%) | 138 (51.5%) | 205 (41%) |  |  |  |
| Medical_Insurance No. (%) |  |  | 500 (100%) | V=0.182 (0.110–0.270) | <0.001 | Chi-square |
| Publicly funded | 34 (14.7%) | 37 (13.8%) | 71 (14.2%) |  |  |  |
| Cooperative medical care | 8 (3.4%) | 6 (2.2%) | 14 (2.8%) |  |  |  |
| Basic medical insurance | 129 (55.6%) | 190 (70.9%) | 319 (63.8%) |  |  |  |
| Self-funded | 61 (26.3%) | 35 (13.1%) | 96 (19.2%) |  |  |  |
| Drinking_History No. (%) |  |  | 500 (100%) | V=0.112 (0.029–0.185) | 0.014 | Chi-square |
| Drinking_History - No | 215 (92.7%) | 262 (97.8%) | 477 (95.4%) |  |  |  |
| Drinking_History – Yes | 17 (7.3%) | 6 (2.2%) | 23 (4.6%) |  |  |  |
| Smoking_History No. (%) |  |  | 500 (100%) | V=0.146 (0.052–0.229) | 0.002 | Chi-square |
| Smoking_History - No | 188 (81.0%) | 245 (91.4%) | 433 (86.6%) |  |  |  |
| Smoking_History – Yes | 44 (19.0%) | 23 (8.6%) | 67 (13.4%) |  |  |  |
| Family_history_of_psychiatric_disorders No. (%) |  |  | 500 (100%) | V=0.302 (0.216–0.375) | <0.001 | Chi-square |
| Family_history —No | 155 (66.8%) | 245 (91.4%) | 400 (80.0%) |  |  |  |
| Family_history —Yes | 77 (33.2%) | 23 (8.6%) | 100 (20.0%) |  |  |  |
| Number_of_Past_High_risk_Factors[M (Q1, Q3)] | 2.0 [1.0, 2.0] | 0.0 [0.0, 0.0] | 1.0 [0.0, 1.0] | ε²=0.488 (0.424–0.570) | <0.001 | Kruskal-Wallis |
| Number_of_Comorbid_Mental_Disorders[M(Q1,Q3)] | 1.0 [1.0, 1.0] | 0.0 [0.0, 1.0] | 1.0 [0.0, 1.0] | ε²=0.5083(0.439–0.574) | <0.001 | Kruskal-Wallis |
| Age_at_First_Onset_of_Disease No. (%) |  |  | 500 (100%) | V=0.191 (0.106–0.270) | <0.001 | Chi-square |
| ≤17y | 32 (13.8%) | 15 (5.6%) | 47 (9.4%) |  |  |  |
| 18-44y | 149 (64.2%) | 212 (79.1%) | 361 (72.2%) |  |  |  |
| 45-59y | 41 (17.7%) | 27 (10.1%) | 68 (13.6%) |  |  |  |
| ≥60y | 10 (4.3%) | 14 (5.2%) | 24 (4.8%) |  |  |  |
| Number_of_episodes[M(Q1,Q3)] | 4.0 [3.0, 5.0] | 1.0 [1.0, 2.0] | 2.0 [1.0, 4.0] | ε²=0.493 (0.428–0.555) | <0.001 | Kruskal–Wallis |
| Number_of_Suicide_Behaviors_after_Diagnosis[M(Q1,Q3)] | 2.0 [0.0, 4.0] | 0.0 [0.0, 0.0] | 0.0 [0.0, 2.0] | ε²=0.481 (0.409–0.558) | <0.001 | Kruskal–Wallis |
| Number_of_Physical_Diseases[M (Q1, Q3)] | 0.0 [0.0, 0.0] | 0.0 [0.0, 0.0] | 0.0 [0.0, 0.0] | ε²=0.039 (0.013–0.076) | <0.001 | Kruskal–Wallis |
| Antidepressant_categeries No. (%) |  |  | 500 (100%) | V=0.200 (0.151–0.285) | 0.003 | Fisher exact |
| SSRIs | 125 (53.9%) | 183 (68.3%) | 308 (61.6%) |  |  |  |
| SNRIs | 44 (19.0%) | 50 (18.7%) | 94 (18.8%) |  |  |  |
| NaSSA | 3 (1.3%) | 2 (0.7%) | 5 (1.0%) |  |  |  |
| SARI | 29 (12.5%) | 15 (5.6%) | 44 (8.8%) |  |  |  |
| Melatonin receptor agonists | 5 (2.2%) | 1 (0.4%) | 6 (1.2%) |  |  |  |
| Aminoketone antidepressants | 8 (3.4%) | 9 (3.4%) | 17 (3.4%) |  |  |  |
| Fixed-dose combination antidepressant | 16 (6.9%) | 7 (2.6%) | 23 (4.6%) |  |  |  |
| Multireceptor-acting antidepressant | 2 (0.9%) | 1 (0.4%) | 3 (0.6%) |  |  |  |
| Degree_of_Improvement_in_Depressive_Symptoms No. (%) |  |  | 500 (100%) | V=0.189 (0.114–0.281) | 0.002 | Fisher exact |
| Worse than before | 2 (0.9%) | 1 (0.4%) | 2 (0.4%) |  |  |  |
| No improvement | 19 (8.2%) | 11 (4.1%) | 26 (5.2%) |  |  |  |
| Mild improvement | 71 (30.6%) | 61 (22.8%) | 120 (24.0%) |  |  |  |
| Moderate improvement | 89 (38.4%) | 93 (34.7%) | 180 (36.0%) |  |  |  |
| Significant improvement | 51 (22.0%) | 102 (38.1%) | 172 (34.4%) |  |  |  |
| Different_organ_systems_of_AEs No. (%) |  |  | 500 (100%) | V=0.258 (0.223–0.353) | <0.001 | Fisher exact |
| Digestive system | 69 (29.7%) | 138 (51.5%) | 207 (41.4%) |  |  |  |
| Cardiovascular system | 40 (17.2%) | 37 (13.8%) | 77 (15.4%) |  |  |  |
| Respiratory system | 4 (1.7%) | 2 (0.7%) | 6 (1.2%) |  |  |  |
| Nervous system | 39 (16.8%) | 36 (13.4%) | 75 (15.0%) |  |  |  |
| Reproductive system | 7 (3.0%) | 5 (1.9%) | 12 (2.4%) |  |  |  |
| Eye/ear/nose/throat | 1 (0.4%) | 4 (1.5%) | 5 (1.0%) |  |  |  |
| Behavioral toxicity and sleep disorder | 41 (17.7%) | 26 (9.7%) | 67 (13.4%) |  |  |  |
| Metabolic system | 18 (7.8%) | 11 (4.1%) | 29 (5.8%) |  |  |  |
| Others 1^a^ | 6 (2.6%) | 6 (2.2%) | 12 (2.4%) |  |  |  |
| Others 2^b^ | 6 (3.0%) | 3 (1.1%) | 10 (2.0%) |  |  |  |
| Severity_of_AEs No. (%) |  |  | 500 (100%) | V=0.399 (0.326–0.489) | <0.001 | Fisher exact |
| Suspicious or mild | 1 (0.4%) | 6 (2.2%) | 7 (1.4%) |  |  |  |
| Mild | 95 (40.9%) | 208 (77.6%) | 303 (60.6%) |  |  |  |
| Moderate | 107 (46.1%) | 46 (17.2%) | 153 (30.6%) |  |  |  |
| Severe | 29 (12.5%) | 8 (3.0%) | 37 (7.4%) |  |  |  |
| Discontinue No. (%) |  |  | 500 (100%) | V=0.604 (0.539–0.675) | <0.001 | Chi-square |
| Discontinue – No | 87 (37.5%) | 253 (94.4%) | 340 (68.0%) |  |  |  |
| Discontinue - Yes | 145 (62.5%) | 15 (5.6%) | 160 (32.0%) |  |  |  |

^a^ Others 1 include 3 psychomotor slowing,2 peripheral edema,2 asthenia with falls,2 restless-legs syndrome,1 derealization,1 formication and 1 pyrexia, data are derived from the "Other" category in Table S2 (Adverse Events reference table), encompassing adverse event types not included among the first 11 organ system categories, which patients self-list by name when completing Table S3 (Antidepressant Adverse Event Questionnaire).

**^b^** Others 2 include 3 Urinary system AEs, 3 Skin AEs, and 4 Hematologic system AEs, derived by collapsing levels with frequencies less than 5 during data preprocessing.

Effect sizes are reported as Cramér's V for categorical variables and epsilon-squared (ε²) for continuous variables, with 95% confidence intervals estimated via bootstrapping. q values represent significance levels after False Discovery Rate (FDR) correction using the Benjamini–Hochberg procedure. As all variables contributed to cluster construction, between-group differences are algorithmically inherent; thus, effect sizes and q values are presented solely to quantify the magnitude of differences across subtype profiles.
